# Analysis of Purified Pancreatic Islet Beta and Alpha Cell Transcriptomes Reveals 11β-Hydroxysteroid Dehydrogenase (Hsd11b1) as a Novel Disallowed Gene

**DOI:** 10.3389/fgene.2017.00041

**Published:** 2017-04-10

**Authors:** Timothy J. Pullen, Mark O. Huising, Guy A. Rutter

**Affiliations:** ^1^Section of Cell Biology and Functional Genomics, Department of Medicine, Imperial College LondonLondon, UK; ^2^Department of Neurobiology, Physiology, and Behavior, College of Biological Sciences, University of California, Davis, DavisCA, USA

**Keywords:** Hsd11b1, glucocorticoids, islets of langerhans, beta cells, alpha cells, diabetes mellitus, disallowed genes

## Abstract

We and others have previously identified a group of genes, dubbed “disallowed,” whose expression is markedly lower in pancreatic islets than in other mammalian cell types. Forced mis-expression of several members of this family leads to defective insulin secretion, demonstrating the likely importance of disallowance for normal beta cell function. Up to now, transcriptomic comparisons have been based solely on data from whole islets. This raises the possibilities that (a) there may be important differences in the degree of disallowance of family members between beta and other either neuroendocrine cells; (b) beta (or alpha) cell disallowed genes may have gone undetected. To address this issue, we survey here recent massive parallel sequencing (RNA-Seq) datasets from purified mouse and human islet cells. Our analysis reveals that the most strongly disallowed genes are similar in beta and alpha cells, with 11β-hydroxysteroid dehydrogenase (*Hsd11b1)* mRNA being essentially undetectable in both cell types. The analysis also reveals that several genes involved in cellular proliferation, including *Yap1* and *Igfbp*4, and previously assumed to be disallowed in both beta and alpha cells, are selectively repressed only in the beta cell. The latter finding supports the view that beta cell growth is selectively restricted in adults, providing a mechanism to avoid excessive insulin production and the risk of hypoglycaemia. Approaches which increase the expression or activity of selected disallowed genes in the beta cell may provide the basis for novel regenerative therapies in type 2 diabetes.

## Introduction

Pancreatic beta cells detect small fluctuations in circulating glucose levels by coupling oxidative metabolism to the regulation of ATP-sensitive K^+^ channels, and consequently to Ca^2+^ influx (Rutter et al., 2015). The loss or dysfunction of beta cells contributes to all forms of diabetes mellitus, a disease affecting more than 400 m individuals worldwide^1^.

Studies in the early 1990s (Sekine et al., 1994) revealed that two “housekeeping” genes, *LDHA* and *SLC16A1* (Monocarboxylate transporter-1, MCT-1), which are abundant in essentially all mammalian cell types and permit vigorous glycolytic flux during anaerobosis, are expressed at vanishingly low levels in beta cells. Subsequent studies by ourselves (Pullen et al., 2010; Pullen and Rutter, 2013) and others (Thorrez et al., 2011; Lemaire et al., 2016) have provided a list of ∼60 genes which are selectively disallowed in these cells, of which there is general consensus on a list of ∼11 genes (Pullen and Rutter, 2013). Re-expression of *Slc16a1* or *Ldha* (Zhao and Rutter, 1998; Ishihara et al., 1999; Ainscow et al., 2000; Pullen et al., 2012) as well as the acyl-CoA thioesterase, *Acot7* (Martinez-Sanchez et al., 2016) in the beta cell leads to defects in insulin secretion, suggesting that the silencing of these genes in beta cells is likely to be functionally relevant.

Previous studies to identify islet disallowed genes have, however, analyzed whole islet transcriptome data (Pullen et al., 2010; Thorrez et al., 2011). Because islets are composed of multiple cell types (Elayat et al., 1995), this has not given a clear picture for any one cell type: the possibility consequently exists that certain genes may be less “disallowed” in the less abundant islet endocrine cells (notably alpha and delta) than in beta cells.

It has therefore been of interest to explore this question using datasets recently made available from highly purified islet cell types (Benner et al., 2014; Adriaenssens et al., 2016; DiGruccio et al., 2016), as well as our own, previously unpublished data. With this goal in mind, we have used a similar strategy to previous analyses but taking advantage of the increased dynamic range of RNA-Seq and the purified cell type datasets to reveal a more detailed insight of genes disallowed alpha and beta cells.

While we confirm that many previously identified islet disallowed genes are indeed disallowed in both alpha and beta cells, we also reveal a number of genes which are expressed at a far lower level in beta cells and whole islets. Strikingly, 11β-hydroxysteroid dehydrogenase (*Hsd11b1*) a critical enzyme for the conversion of the inactive precursor of corticosterone, 11-dehydrocorticosterone (11-DHC) (Seckl and Walker, 2001), is found to be remarkably weakly expressed in both beta and alpha cells, as well as in delta cells. This suggests that previous findings demonstrating actions of 11-DHC on insulin secretion (Davani et al., 2000) may reflect a requirement for non-beta cells in the activation of this molecule.

We also note that genes involved in cell proliferation (e.g., *Yap1*, *Igfbp4*) are more selectively inactivated in beta than alpha cells. This is consistent with an evolutionarily driven mechanism to suppress the proliferation of beta cells in adults and thus a risk of life-threatening hypoglycaemia.

## Materials and Methods

### Identification of Disallowed Genes in Adult Mouse Tissues and Purified Cells

RNA-Seq datasets for a range of normal mouse tissues were obtained from public repositories and our own data. These included: 10 datasets for FACS-purified alpha cells from three studies (Benner et al., 2014; Adriaenssens et al., 2016; DiGruccio et al., 2016); 14 FACS-purified beta cell datasets from four studies (Benner et al., 2014; Adriaenssens et al., 2016; DiGruccio et al., 2016; E-MTAB-2266); and 15 islet datasets from our own and other studies (Kone et al., 2014; GSE90531). Non-islet tissue datasets covering brain, heart, kidney, liver, lung, spleen, and thymus were included for comparison. Dispersed, FACS-purified cells contain many differences to dissected tissues including lack of blood and endothelial cells. To control for these differences, eight datasets of FACS-purified hepatocytes were included. Islet, alpha, and beta cell data were derived from data series with GEO or ArrayExpress accession numbers: GSE54973, E-MTAB-2266, GSE80673, GSE76017, GSE90531, and E-MTAB-2791. Non-islet tissue and cell data were derived from data series with accession numbers: GSE36025, GSE74747, GSE65207, and GSE68806.

Where data were in paired-end format, the second read was discarded for each fragment to allow a more direct comparison with the single-ended data. Reads were mapped to the mouse genome (GRCm38) using HiSat2 (Kim et al., 2015) and annotated transcripts quantified with featureCounts (Liao et al., 2014). Differential expression analysis was performed using DESeq2 (Love et al., 2014). Alpha cell, beta cell, and islet data were then subjected to pairwise comparisons against each non-islet tissue and cell type. There is an acknowledged problem that detecting differential expression by significance alone can result in the selection of genes with small but consistent changes which are of questionable biological significance while excluding larger but more variable changes. In an attempt to overcome this, the fold-change and significance were combined into a single metric, the π-value (Xiao et al., 2014). The π-values for each pairwise comparison were combined where the direction of change was consistent (e.g., genes whose expression was higher or lower in beta cells than all other non-islet datasets) by taking the π-value closest to zero (i.e., least combined significance and fold-change) for each gene. Genes were ranked by π-value allowing the most consistently under- and over-expressed genes to be identified at both ends of the resulting list. Since the number of datasets included for alpha cells, beta cells, and islets was not consistent, this is likely to have affected the number of differentially expressed genes detected in each case. For this reason, we have concentrated on comparing the ‘most disallowed’ genes at the top of the ranked gene lists in these cell types rather than using a significance/fold-change threshold to define disallowance.

### Cluster Analysis

The top 50 disallowed genes from alpha and beta cells were combined and hierarchical clustering performed on the basis of fold-change in gene expression in each tissue/cell type relative to islets. Weighted gene co-expression network analysis (WGCNA) was also performed on variance-stabilizing transformed count data exported from DESeq2 (Langfelder and Horvath, 2008). Functional characterization of gene lists using Gene Ontology (GO) Biological Process and PANTHER protein class was performed through the PANTHER website (Mi et al., 2017).

### Developmental Regulation of Disallowed Genes

Transcriptomes of FACS-purified beta cells at different peri- and post-natal maturation stages were generated using the mIns1-H2b-mCherry reporter line (Benner et al., 2014) (Jax # 028589). Breeders homozygous for mIns1-H2b-mCherry were crossed to wild-type C57bl6 mates to ensure offspring were uniformly hemizygous for the mCherry reporter. Islets from single litters (a mix of male and female is expected) were pooled for each sample to obtain sufficient material. Pooled islets were dissociated, sorted and collect in Trizol for RNA isolation and library construction. Each time point was collected in the morning and FACS-sorted at the conclusion of the islet prep the same day. Each time point was done in duplicate or triplicate. Data are deposited under GEO accession number GSE88779.

### Gene Expression Association with Type 2 Diabetes Status in Human Islets

To investigate whether the expression of the human orthologues of these genes are altered during the progression of type 2 diabetes, we exploited RNA-Seq data on pancreatic islets isolated from 89 human donors (Fadista et al., 2014). Gene expression was quantified using the pipeline described above using the GRCh37 genome, and differential expression relative to diabetes status determined by DESeq2. The top 20 beta cell disallowed genes were investigated for a significant association between expression and diabetes status.

## Results

**FIGURE 1:**
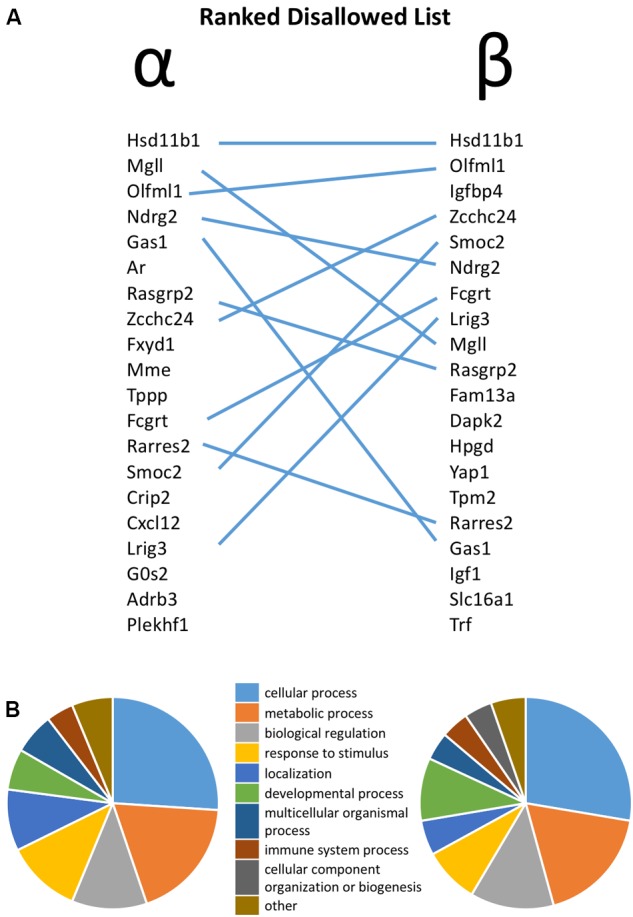
**(A)** Ranked list of the top 20 most disallowed (selectively down-regulated) genes in pancreatic alpha and beta cells, with overlap between the two lists highlighted by lines. **(B)** Functional annotation of the top 50 disallowed gene in each cell type on the basis of Gene Ontology (GO) Biological Process.

Once the datasets were assembled, the analysis yielded the genes which were most enriched and most disallowed in alpha and beta cells (**Figure 1A**). The results were ranked by the π-value which combined both the log fold change and adjusted *p*-value into a single metric. It should be noted that our analysis is based on pairwise comparisons between the islet cell type and each of the non-islet tissues, and the smallest π-value from each of these comparisons is used for each gene. Since this analysis is based on the smallest π-value from comparisons with each other tissue it identifies genes which are consistently over- or under-represented in a particular cell type, rather than those with the largest median difference.

The similarity between the 20 genes most consistently repressed in alpha and beta cells, relative to non-islet tissues, is notable. Thus, 11 of these genes are common between the two lists. Functional classification of the top 50 disallowed genes in each cell type again revealed the similarity between the two lists, with ‘cellular process,’ ‘metabolic process’ and ‘biological regulation’ being the top three GO Biological Process terms in both lists (**Figure 1B**).

**FIGURE 2:**
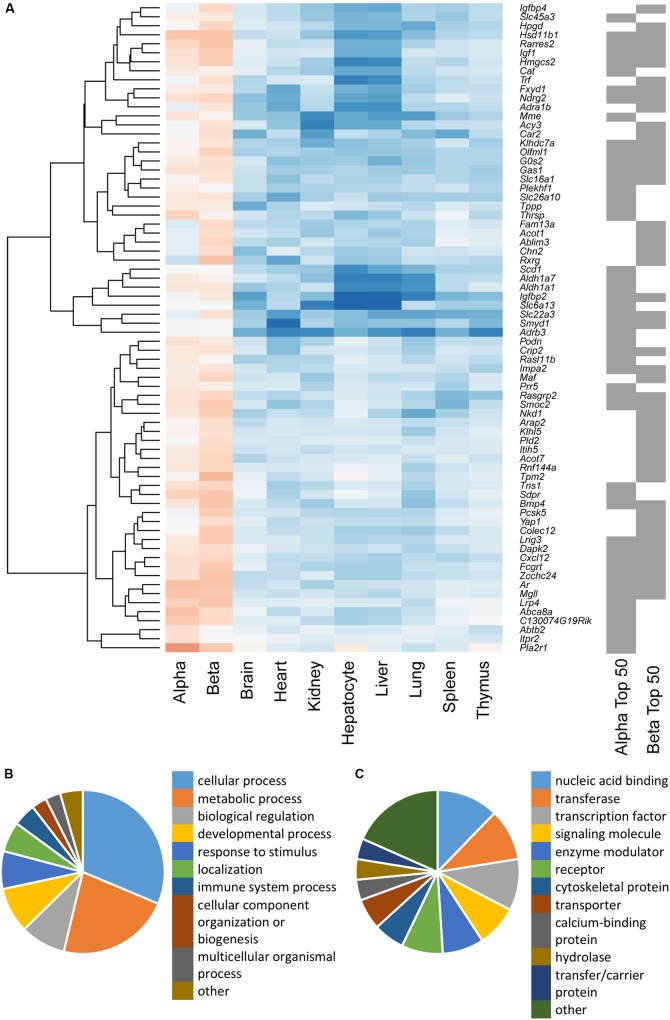
**(A)** Hierarchical cluster analysis of a combined list of the top 50 disallowed genes from both alpha and beta cells. Membership of the top 50 alpha and/or beta cell list is indicated by the gray bars on the right. Heatmap shading denotes fold-change in gene expression relative to islets, with blue representing up- and red down-regulation. A cluster of 50 genes selectively repressed in beta but not alpha cells was identified by weighted gene co-expression network analysis (WGCNA), and genes were functionally annotated on the basis of Biological Process **(B)** and Protein Class **(C)**.

To study the similarities between the lists for the two cell types, the top 50 disallowed genes for each were merged and clustered on the basis of differential expression across all cell/tissue types (**Figure 2A**). While 27 of these genes were shared between the two cell types, the differences between the lists were equally interesting. One cluster contained five genes which were disallowed selectively in beta cells, but not alpha cells (*Fam13a, Acot1, Ablim3, Chn2, Rxrg*). The genes in this cluster exhibit lower expression in purified beta cells than in whole islets, and higher expression in alpha cells. A smaller cluster of three genes (*Pcsk5, Yap1, Colex12*) has a similar expression pattern, whereas the majority of the other beta cell disallowed genes also had low expression in alpha cells.

We sought to further investigate the differences in selectively repressed genes between alpha and beta cells using WGCNA of normalized count data from all the tissue/cell types. This approach identified a single module, containing 50 genes, which was significantly down-regulated in beta cells (*p* = 3 × 10^-17^) and islets (*p* = 2 × 10^-12^) but not alpha cells (*p* = 0.3; **Supplementary Table S1**). This module included most of the genes in the clusters described above. Searching for enrichment of GO terms revealed the enzyme-linked receptor signaling pathway (*p* = 0.023). This observation provides insights into possible differences in the proliferative capacity of alpha and beta cells.

Functional classification of the genes within this module showed that many were associated with metabolic processes (**Figure 2B**). A preponderance of nucleic acid binding, transcription factor and signaling molecules among the protein classes (**Figure 2C**) also indicates that selective silencing of this module in beta cells may contribute to the regulation of beta cell identity.

**FIGURE 3:**
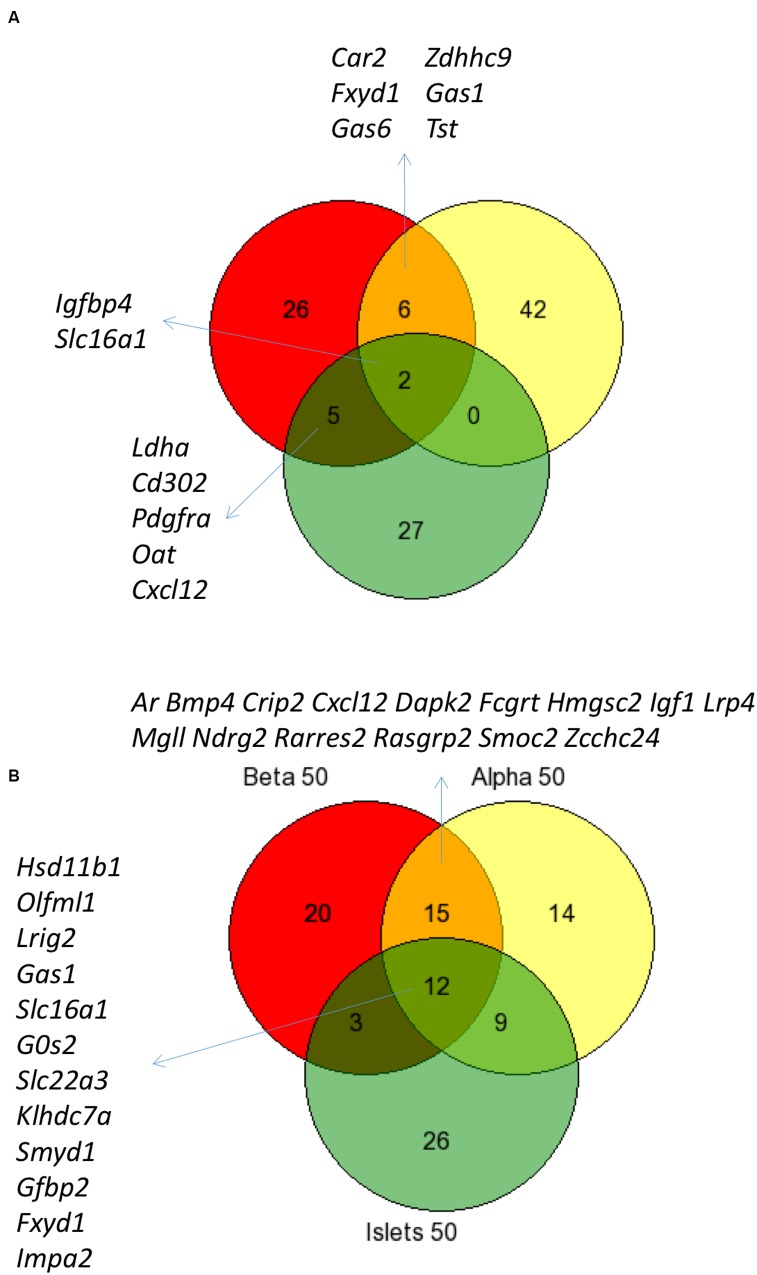
**Comparison of disallowed gene expression in isolated mouse islet cells *versus* intact islets**. Venn diagram showing the overlap between the top 50 disallowed islet genes from this study (Yellow) with lists from previous studies by Pullen et al. (2010; Red) and Thorrez et al. (2011; Green) **(A)**. The overlap between the top 50 disallowed genes from islets (Green), alpha (Yellow), and beta cells (Red) is also shown **(B)**.

**Figure 3** shows the intersection of data between previous analyses and the current analysis of islet disallowed genes (A) and between the different cell types and islets (B) and reveals that while there is considerable overlap between these datasets, we also noted genes not previously classed as disallowed.

**FIGURE 4:**
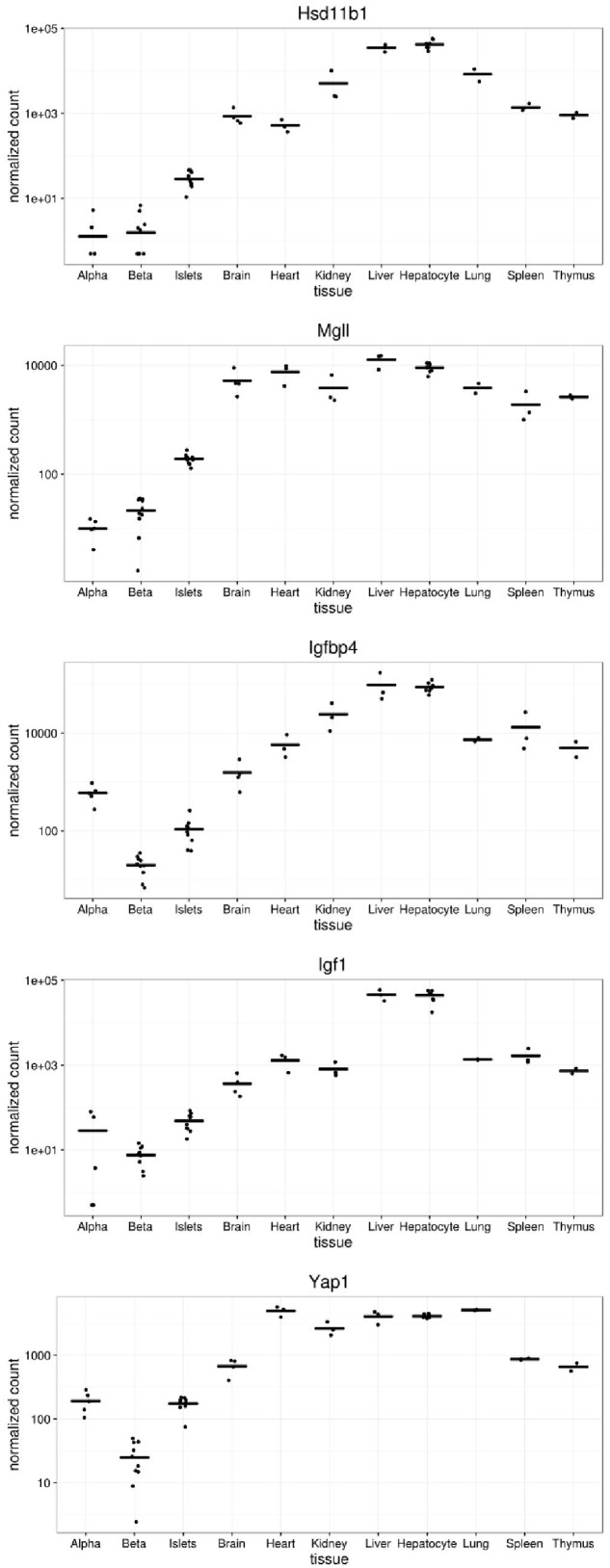
**Expression of selected disallowed genes across different mouse tissues**. Gene expression is presented in normalized read counts per gene along with mean expression level across all tissues are shown for some of the genes highlighted in this study.

We next compared the levels of expression of five of the genes disallowed in alpha and/or beta cells (**Figure 4**). Of these, the most dramatically disallowed is *Hsd11b1* with nearly a 1000-fold gradient existing between expression in brain versus purified alpha or beta cells, where mRNA levels were at or below the level of detection (<10 normalized counts). Relatively abundant expression in other islet cell types presumably explains its considerably higher expression in isolated islets.

Interestingly, both *Igfbp4* and *Yap1* are approximately one order of magnitude more highly expressed in alpha than beta cells, indicating that the disallowance of these genes is likely to be more relevant to the beta cell phenotype than that of alpha cells.

**FIGURE 5:**
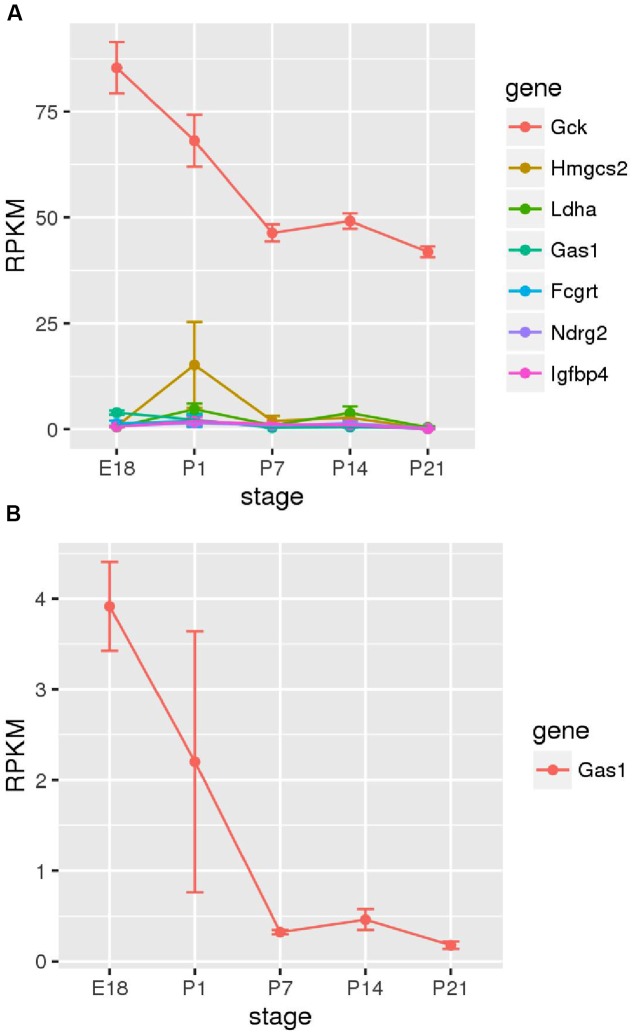
**Changes in disallowed gene expression during post-natal development**. Expression of disallowed genes in purified insulin-positive cells from pre- and post-natal mice is shown along with the expression of glucokinase (*Gck*) for comparison. The six genes exhibiting the highest expression during this period are shown **(A)**, and *Gas1* is also shown as one of the few genes exhibiting a consistent down-regulation during this period **(B)**.

Next, we sought to determine the time points at which members of the family of disallowed genes become inactivated in beta cells through analyzing RNA-Seq data from purified beta cells at various developmental stages (**Figure 5**). Strikingly, of those sampled, the majority were already very weakly expressed by embryonic day 18 (E18). *Gas1* provided an exception in that mRNA levels encoding this enzyme declined (albeit from low levels) between E18 and P7.

Finally, we investigated whether increased expression of any of these disallowed genes is associated with type 2 diabetes (**Table 1**). Of the top 20 beta cell disallowed genes, increased expression of six was associated with diabetes with an adjusted *p*-value < 0.1, with *IGF1* showing the most significant association.

**Table 1:** The association of expression of beta cell disallowed genes in human islets with type 2 diabetes status.

Gene	baseMean	LFC	AdjP
*IGF1*	5.87	0.957	0.0185
*MGLL*	442	0.738	0.0357
*CXCL12*	500	0.774	0.0448
*IGFBP4*	4241	0.566	0.0786
*SMOC2*	57	0.718	0.0932
*SLC16A1*	430	0.509	0.0959

*RNA-Seq data from Fadista et al. (2014) were analyzed relative to diabetes status determined by HbA1c levels. The six genes showing associations with adjusted *p* < 0.1 are reported here, and all have positive log fold change (LFC) indicating that type 2 diabetes is associated with increased expression*.

## Discussion

### Comparison of Current Methodology to Previous Studies

The approach taken in this analysis differs from previous analyses in a number of ways, in addition to the use of data from purified cell types rather than isolated pancreatic islets. The largest difference is the use of RNA-Seq rather than microarray data. The principle advantages of RNA-Seq are the higher dynamic range (Zhao et al., 2014) and the fact that quantification is based on the whole length of the gene rather than the limited coverage of probesets. However, in addition to using a different platform, the analysis is also different in the current study.

Previous analyses have concentrated either on the statistical significance of the difference (Thorrez et al., 2011), or on a fold-change threshold (Pullen et al., 2010). The present analysis combined both measures into a π-value with the aim of capturing both highly significant (consistent) changes of a smaller magnitude and less significant (more variable) changes with a greater magnitude. Furthermore, ranking genes by this combined score is arguably more biologically meaningful than ranking by either significance or fold-change alone. We note, however, that demonstration of the relative strengths of this approach versus others will require future biological assessments, e.g., overexpression of candidate genes (Pullen et al., 2012; Martinez-Sanchez et al., 2016).

It is possible to assess the combined effect of these changes by comparing the current analysis of whole islets with previous analyses (**Figure 3**). The overlap between the top 50 islet disallowed genes from this study and the lists of 39 and 40 genes from the Pullen et al. (2010) and Thorrez et al. (2011) papers, respectively, is just two genes: *Slc16a1* and *Igfbp4*. This is reassuring since there is strong evidence that *Slc16a1* is specifically silenced in islets (Zhao et al., 2001), and that mis-expression of this gene in human (Otonkoski et al., 2007) and mouse (Pullen et al., 2012) beta cells causes inappropriate stimulation of insulin secretion in response to pyruvate.

The current list also contains six genes which overlap with the Pullen list but none with the Thorrez list. It is interesting to note that five genes common to both the Pullen and Thorrez lists are not included in the current islet list: *Ldha*, *Cd302*, *Pdgfra*, *Cxcl12*, and *Oat*. Of these, both *Ldha* and *Oat* are within the top 100 of the current list, so while their rank may be altered slightly in the current analysis, there is still strong evidence for their categorization as islet disallowed genes.

*Pdgfra* is an interesting disallowed gene because there is evidence that a gradual lowering in its expression plays a role in the age-dependent decline in beta cell proliferative capacity (Chen H. et al., 2011). However, *Pdgfra* also displays relatively low expression in liver and strikingly low expression in purified hepatocytes. While this does not detract from the important role of *Pdgfra* repression in beta cells, it does exclude it from our strict definition of islet disallowed genes.

### Alpha and Beta Cell Disallowed Genes

The main aim of this study was to identify novel disallowed genes in alpha and beta cells, particularly those whose low expression may have been masked by expression in other islet cell types. When searching for genes specifically expressed in a particular cell type, low levels of contamination with other cell types have relatively little impact because they are unlikely to lead to false negatives and will only occasionally lead to false positives if the level of contamination is high. However, contamination is likely to be more of an issue when searching for cells specifically repressed in one cell type, since the expression in the contaminating cells will be easily detectable over the low background.

A striking feature of the lists of alpha and beta cell disallowed genes is their remarkable similarity. In contrast to enriched genes, which contain few similarities between cell types (Xin et al., 2016), the disallowed genes were notably similar (**Figure 1**). Looking at this aspect of cell identity suggests that alpha and beta cells are more similar than the impression given by looking at highly expressed genes, and is presumably an important aspect of reprogramming from an alpha cell to a beta fate (and vice versa) in some circumstances (Collombat et al., 2009; Thorel et al., 2010).

There is also considerable overlap between alpha cell, beta cell, and islet disallowed gene lists (**Figure 3B**), which includes several previously identified islet disallowed genes: *Slc16a1*, *Fxyd1*, *Ndfg2*, *Gas1*. However, it is particularly interesting to consider a few examples of disallowed genes which are either newly identified or ranked more highly in the current analysis of purified cells.

*Hsd11b1* is the highest ranked beta cell disallowed gene, and second on the alpha cell list. While *Hsd11b1* is also included on the islet disallowed list, the reason for its high rank in the purified cell types becomes apparent from a plot of its expression profile (**Figure 4**). Although the islet expression level is over an order of magnitude lower than any non-islet tissue, expression in alpha and beta cells is a further order of magnitude below this. Indeed, since the DESeq2 tool used in this analysis adds 0.5 to all normalized counts it can be seen from **Figure 4** that no reads mapped to *Hsd11b1* in two alpha cell, and three beta cell samples, and fewer than 10 normalized counts were detected in any alpha or beta cell sample. This level would normally be considered below the threshold of detectable expression, i.e., the gene is essentially silent in both cell types. Of note, although expression of *Hsd11b1* is still very low in islets, the levels detected in the intact micro-organ are much higher than in purified alpha or beta cells. We find no evidence of expression in delta cells (DiGruccio et al., 2016) so this most likely reflects significant *Hsd11b1* expression in other non-endocrine islet cell types (endothelial, pericytes, etc.).

*HSD11B1* encodes an enzyme which converts inert cortisone into active cortisol (or 11-dehydroxycorticosterone to corticosterone in rodents), thus regulating local glucocorticoid action in metabolically active tissues (Seckl and Walker, 2001). Glucocorticoids have long been associated with adverse metabolic effects, including increased insulin resistance. Indeed, steroid-induced diabetes is a significant complication of glucocorticoid therapy (Hwang and Weiss, 2014). *Hsd11b1* is reportedly the major regulator of tissue-specific glucocorticoid effects (Morgan et al., 2014). Overexpression of *Hsd11b1* in either liver (Paterson et al., 2004) or adipose tissue (Masuzaki et al., 2003) renders mice insulin resistant, conversely knockout of this gene protects mice from glucocorticoid-induced insulin resistance (Morgan et al., 2014). In humans *HSD11B1* hyperexpression has been associated with abnormal glucose metabolism and obesity in several studies (Nascimento et al., 2015). Whereas the diabetogenic effects of glucocorticoids have mainly been attributed to increased insulin resistance, there is some evidence that they involve beta cells directly.

Many reports of the effects of glucocorticoids on beta cells have highlighted inhibitory roles such as suppression of both insulin secretion (Chen F. et al., 2011) and cellular proliferation (Colvin et al., 2013) by the synthetic glucocorticoid, dexamethasone. Indeed, it would be adaptive for endocrine cells not to generate cortisol/corticosterone locally as this would preserve their ability to respond to circulating cortisol [derived from hypothalamus pituitary adrenal (HPA) axis activity] without any interference of local 11 HSD activity. After all, GCs are the functional antagonist of insulin and the two are cross-regulatory.

We have previously shown (Huising et al., 2011) that acute dexamethasone stimulation inhibits beta cell GLP1 receptor expression in a glucocorticoid receptor-dependent manner, confirming earlier findings. It is interesting to note that acute restraint stress elicited similar effects in mouse islets after 3 h, and these had normalized after 12 h. This suggests that acute stress transiently attenuates the sensitivity of beta cells to incretins by suppressing the expression of its receptors in order to facilitate the hyperglycemia induced by glucocorticoids. This acute, transient repression of insulin release by glucocorticoids is, in the above context, adaptive. However, in metabolic syndrome, the same mechanisms may rapidly become maladaptive.

However, other studies suggest that glucocorticoids may *stimulate* insulin secretion and/or beta cell survival in certain circumstances. For example, *in vitro* pre-treatment with low concentrations (200 nM) of corticosterone for 18 h increased glucose-stimulated insulin secretion from isolated islets (Hult et al., 2009). Dex also reportedly increased glucose-stimulated insulin secretion in islets isolated from rats that were treated *in vivo* with Dex (Rafacho et al., 2010), although it is difficult to dissociate effects on the islet from other systemic actions. Glucocorticoid pre-treatment also improved the function of transplanted human islets (Lund et al., 2008). Finally, overexpression of *Hsd11b1* is reported to offer some protection against high-fat diet-induced glucose intolerance, through increasing both islet mass and function (Turban et al., 2012). Together, these findings suggest that the anti-inflammatory action of glucocorticoids may enhance beta cell function under certain conditions, although prolonged and/or high level treatment may interfere with both beta cell function and proliferation.

While we demonstrate here strikingly low expression of *Hsd11b1* in both alpha and beta cells, there are apparently contradictory reports in the literature concerning the expression of this gene in beta cells and islets. Schmid et al. (2011) reported HSD11B1 expression at the protein level throughout rat islets and in INS-1 insulinoma cells, although this finding is sharply refuted in a commentary by Liu et al. (2011) who question the specificity of the antibody used. These authors also refer to their previous paper (Chowdhury et al., 2015) reporting *HSD11B1* expression in glucagon-positive cells of wild-type mouse islets by immunofluorescence, and in islets by Western (immuno-) blotting. There are further reports of HSD11B1 detection in mouse islets after treatment with high-fat diet (Turban et al., 2012) or in *ob/ob* mice (Davani et al., 2000). It is difficult to reconcile these reports with the very low levels detected here in both purified alpha and beta cells by RNA-Seq. Firstly, there are clearly questions over the specificity of some of the antibodies used in the previous studies. Secondly, we do detect substantially higher expression in islets than in purified alpha or beta cells, and previous use of qPCR and Western blotting were mainly performed on isolated islets rather than purified cells. Our results suggest that the *Hsd11b1* expression detected in whole islets comes almost exclusively from islet cell types other than alpha and beta cells. Of course, since RNA-Seq measures the transcript level it is possible that is does not accurately reflect the protein level. However, it is difficult to envisage how such a large discrepancy could occur. Finally, the cells used for the RNA-Seq analysis were all from mice on normal diet, and it is possible that *Hsd11b1* expression is induced under certain conditions such as high-fat diet.

Interestingly, *HSD11B1* levels were undetectable in 4/7 purified human beta cell preparations sampled by Blodgett et al. (2015) and at <1 RPKM in the remaining three samples. *HSD11B1* mRNA was also absent in 2/7 purified human alpha cell samples, and at around 1 RPKM in the other five. The latter data indicate that the enzyme is also likely to be disallowed in both human beta and alpha cells, as well as in rodent cells. In each species this presumably provides a mechanism for “protecting” these cell types from the effects of 11-DHC, or ensuring that the actions of the precursor on the secretory function of these cells (Davani et al., 2000) is via the presence of this enzyme in neighboring islet cell types, as discussed above.

*Mgll* encodes monoglyceride lipase which is responsible for the unregulated hydrolysis of monoacylglycerides (MAG) in adipocytes and other tissues (Karlsson et al., 1997). Two other lipases, Adipose Triglyceride Lipase (ATGL) and Hormone Sensitive Lipase (HSL/LIPE) are both expressed in beta cells and have been proposed to stimulate insulin secretion through the production of lipid-derived coupling factors including MAG (Fex et al., 2009; Attané et al., 2016). By degrading this MAG, it is likely that expression of MGLL would remove this coupling factor and decrease insulin secretion. Interestingly, both *Mgll* and *Lipe* are dysregulated in beta cells null for *PGC-1*α/β. Knockout of these PPARγ co-activators impairs fatty-acid potentiated insulin secretion and was accompanied by down-regulation of *Lipe* and up-regulation of *Mgll*. It is tempting to speculate that maintained expression of *Atgl* and *Lipe*, along with suppression of *Mgll* are necessary to allow MAG to act as a coupling factor. It is interesting to note that overexpression of *Acot7*, another disallowed gene involved in lipid metabolism, impairs glucose-stimulated insulin secretion, although this appeared to be through increased ATP consumption rather than decreased MAG levels (Martinez-Sanchez et al., 2016).

Expression of *PGC-1*α is reduced in the islets from subjects with type 2 diabetes (Ling et al., 2008), and this is paralleled by a significant increase in *Mgll* expression in islets from diabetic subjects (**Table 1**). Although it is unclear whether there is a direct link between *PGC-1*α and *Mgll* expression, the dysregulation of lipid metabolism associated with these changes could contribute to the impaired beta cell function in type 2 diabetes.

Numerous studies have reported the pro-proliferative role of IGF-1 on beta cells (reviewed in Stewart et al., 2015), and exogenous IGF-1 treatment has been shown to increase beta cell proliferation (Sieradzki et al., 1988). However, the effects of locally produced IGF-1 may differ from those stimulated by raised circulating levels of the growth factor. Beta cell-specific overexpression of IGF-1 increased the survival and proliferation of beta cells after challenge with multiple low dose streptozotocin-treatment (George et al., 2002). Since this overexpression had little effect on systemic serum levels of IGF-1 it presumably acted in an autocrine/paracrine manner.

The action of IGF-1 is modulated by a family of IGF binding proteins which regulate its bioavailability. *Igfbp4* is strongly disallowed in beta cells, and *Igfbp2* is also within the top 50 disallowed genes for both alpha and beta cells. IGFBP4 is generally considered to have a negative regulatory role on IGF-1, for example, both *IGFBP4* and *IGFBP2* were found to be down-regulated through promoter hypermethylation in lung carcinomas (Sato et al., 2006). In addition to binding to IGF-1, thus preventing it binding to IGF receptors, IGFBP4 has also been reported to act independently of IGF (reviewed in Durai et al., 2006). However, there is growing evidence that IGFBP4 may differentially regulate proliferation in different contexts. Transfecting primary renal cancer cell lines with *IGFBP4* increased proliferation, invasion, and motility, whereas knocking down the gene in metastatic renal cancer cell lines decreased proliferation (Ueno et al., 2011).

The complexity of its varying roles in other tissues makes it difficult to predict what the effect of *Igfbp4* overexpression in islets would be. It also makes it difficult to suggest an adaptive reason for its relative down-regulation in beta cells, and how this might interact with the disallowance of *Igf1*. It is notable that increased expression of both *IGF1* and *IGFBP4* in islets show some association with type 2 diabetes (**Table 1**). Of the top 20 beta cell disallowed genes, *IGF1* showed the strongest association with diabetes status (*p* = 0.0185), whereas the association with increased *IGFBP4* was less strong (*p* = 0.0786). Future studies, in which *Igfbp1* is selectively over-expressed in beta cells, will be needed to solve this conundrum.

*Yap1* is a potent driver of cell growth via the Hippo pathway (Hansen et al., 2015). Interestingly, a survey of human adult alpha and beta cell expression data reveals levels of ∼1 RPKM for *YAP1* in both cell types, albeit with slightly lower levels in beta cells (Blodgett et al., 2015). Nonetheless, in mouse cells, we found that *Yap1* and *Pdgfra* were selectively repressed in beta cells, consistent with a lower proliferative capacity of these cells relative to alpha cells.

We therefore suspect that its suppression in beta cells provides a mechanism to inhibit beta cell expansion, and hence fatal hypoglycaemia. The absence of *Yap1*, *Igf1* and potentially *Igfbp4* may also contribute to the low proliferative index of beta cells in rodents but, more particularly in man, when cell division is barely detectable after the age of ∼21 (Cnop et al., 2010). Moreover, this, alongside the absence of *Pdgfra* (see above) may equally restrict compensatory beta cell growth in times of metabolic need including insulin resistance provoked by diet, aging or pregnancy. Thus, mechanisms designed to increase the expression or these receptors, or to engage their downstream signaling pathways, may provide new approaches toward regeneration of beta cell mass in type 2 diabetes.

### Developmental Regulation of Expression

It is interesting to investigate at what stage in the developing islet expression of these genes is down-regulated relative to other tissues. It has previously been reported in developing rat islets that *Ldha* and *Slc16a1* expression peaked at post-natal day 1 before reducing to near adult levels by P21 (Thorrez et al., 2011). The current investigation of purified insulin-positive cells from mice at stages E18 – P21 did not reveal consistent down-regulation of disallowed genes during this period (**Figure 5A**). Most of the beta cell disallowed genes exhibited low expression (<5 RPKM) by E18 and showed little clear change during the post-natal period. One exception was *Gas1* which did show considerable down-regulation between E18 and P7. However, this pattern did not extend across the disallowed genes investigated. One possible explanation for these differing results is that Thorrez et al. (2011) measured expression in whole islets versus the purified insulin-positive cells reported here. It is therefore possible that the down-regulation observed in whole islets represents increases in the proportion of beta (and other endocrine) cells in the developing islet, whilst the expression of disallowed genes within these remains unchanged during this period, as demonstrated experimentally here.

## Conclusion

The present report provides several novel insights. Notably, we identify a novel member of the beta cell disallowed group, *Hsd11b1*. Secondly, we reveal that assumptions as to the expression of established members of the family, *Yap1* and *Igf4bp1*, as being disallowed in the alpha as well as the beta cell, were misplaced. We further identify a module of 50 genes, including *Yap1*, which are selectively down-regulated in beta but not alpha cells. Our findings may provide the basis of new approaches toward improving beta cell identity and function in type 2 diabetes.

## Author Contributions

TP and GR conceived and planned the study. TP and MH selected and analyzed data. TP, GR, and MH wrote and edited the manuscript.

## Conflict of Interest Statement

The authors declare that the research was conducted in the absence of any commercial or financial relationships that could be construed as a potential conflict of interest.

## Footnotes

1 http://www.idf.org

## Supplementary Material

TABLE S1 **Selectively down-regulated beta cell gene module**.
